# Perioperative therapy for limited-stage small cell esophageal carcinoma: a retrospective cohort study

**DOI:** 10.1093/oncolo/oyaf264

**Published:** 2025-08-26

**Authors:** Jun Peng, Jin-Feng Chen, Yi Wang, Jiang Zhu, Jin-Tao He, Qi Lai, Xue-Feng Leng, Ying-Zhao Huang, Xiang Zhuang

**Affiliations:** Department of Thoracic Surgery, Sichuan Clinical Research Center for Cancer, Sichuan Cancer Hospital & Institute, Sichuan Cancer Center, University of Electronic Science and Technology of China, Chengdu 610041, China; Department of Head and Neck Surgery, Sichuan Clinical Research Center for Cancer, Sichuan Cancer Hospital & Institute, Sichuan Cancer Center, University of Electronic Science and Technology of China, Chengdu 610041, China; Department of Radiation Oncology, Radiation Oncology Key Laboratory of Sichuan Province, Sichuan Clinical Research Center for Cancer, Sichuan Cancer Hospital & Institute, Sichuan Cancer Center, University of Electronic Science and Technology of China, Chengdu 610041, China; Department of Thoracic Surgery, Sichuan Clinical Research Center for Cancer, Sichuan Cancer Hospital & Institute, Sichuan Cancer Center, University of Electronic Science and Technology of China, Chengdu 610041, China; Department of Thoracic Surgery, Sichuan Clinical Research Center for Cancer, Sichuan Cancer Hospital & Institute, Sichuan Cancer Center, University of Electronic Science and Technology of China, Chengdu 610041, China; Department of Thoracic Surgery, Sichuan Clinical Research Center for Cancer, Sichuan Cancer Hospital & Institute, Sichuan Cancer Center, University of Electronic Science and Technology of China, Chengdu 610041, China; Department of Thoracic Surgery, Sichuan Clinical Research Center for Cancer, Sichuan Cancer Hospital & Institute, Sichuan Cancer Center, University of Electronic Science and Technology of China, Chengdu 610041, China; Department of Thoracic Surgery, Sichuan Clinical Research Center for Cancer, Sichuan Cancer Hospital & Institute, Sichuan Cancer Center, University of Electronic Science and Technology of China, Chengdu 610041, China; Department of Thoracic Surgery, Sichuan Clinical Research Center for Cancer, Sichuan Cancer Hospital & Institute, Sichuan Cancer Center, University of Electronic Science and Technology of China, Chengdu 610041, China

**Keywords:** limited-stage small cell esophageal carcinoma, perioperative therapy, long-term survival outcomes, postoperative complications

## Abstract

**Background:**

The optimal treatment strategy for limited-stage small cell esophageal carcinoma (LS-SCEC) remains uncertain. This study aimed to retrospectively evaluate the efficacy of perioperative therapy in these patients.

**Materials and Methods:**

Between June 2005 and November 2022, 156 patients with LS-SCEC who underwent esophagectomy were included in this study. The primary endpoint was overall survival (OS), and secondary endpoints included disease-free survival (DFS) and postoperative complications. Prognostic factors were analyzed using univariate and multivariate Cox regression models, as well as propensity score matching.

**Results:**

Of the 156 patients, 33 (21.2%) patients received neoadjuvant chemotherapy, 76 (48.7%) underwent adjuvant chemotherapy, and 13 (8.3%) received adjuvant chemoradiotherapy. Post-surgery, 33 (21.2%) patients were classified as stage I, 40 (25.6%) as stage II, 61 (39.1%) as stage III, and 22 (14.1%) as stage IV. The median OS was 21.0 months, with 5-year OS and DFS rates of 25.9% and 21.9%, respectively. Multivariate analysis identified clinical T stage (*P* = .049), neural invasion (*P* = .002), N stage (*P* = .006), and adjuvant therapy (*P* = .001) as independent prognostic factors. Neoadjuvant chemotherapy significantly improved OS, with a 5-year OS rate of 37.7% compared to 0.0% in the non-neoadjuvant chemotherapy group (*P* < .001). Across the cohort, adjuvant therapy enhanced both OS and DFS.

**Conclusions:**

Perioperative therapy is crucial for LS-SCEC management. Neoadjuvant chemotherapy improves OS in patients with LS-SCEC. Adjuvant chemotherapy is recommended almost for all patients with LS-SCEC.

Implications for practice This study includes the largest cohort of LS-SCEC patients who underwent surgery at a single center, providing a comprehensive analysis of long-term outcomes after perioperative treatment. PSM analyses demonstrated that neoadjuvant chemotherapy significantly improved OS in LS-SCEC patients, while adjuvant therapy enhanced both OS and DFS. These results highlight the importance of perioperative treatment for LS-SCEC patients. Neoadjuvant chemotherapy improves OS in patients with LS-SCEC. Adjuvant chemotherapy is recommended almost for all patients with LS-SCEC.

## Introduction

Small cell esophageal carcinoma (SCEC) is a rare cancer, comprising only 0.4%-2% of all esophageal malignancies.^1^^,^^2^ Similar to small cell lung cancer (SCLC), SCEC is highly aggressive, with 40%-60% of cases presenting with distant metastases at diagnosis, resulting in a poor prognosis.^3^^,^^4^

Due to the rarity of SCEC, conducting prospective clinical trials is challenging. Current therapeutic strategies for SCEC, which combine locoregional treatment and systemic therapy, are primarily adapted from SCLC treatment guidelines.^4^ For patients with extensive-stage disease, chemotherapy combined with radiotherapy remains the standard treatment.^3^^,^^5^ However, the optimal strategy for limited-stage (LS) SCEC remains controversial.^6^ Several studies have indicated that surgical resection for limited disease may improve patient outcomes.^3^^,^^5–7^ Nevertheless, most patients undergoing surgery alone experience rapid systemic recurrence.^8^ Consequently, many institutions have adopted a multidisciplinary approach, integrating local therapy (surgery or radiotherapy) with systemic therapy.

We hypothesized that a multidisciplinary approach, incorporating both systemic therapy and surgery, would achieve superior outcomes for resectable LS-SCEC. This study aimed to describe the characteristics of patients receiving different treatments and compare outcomes between those treated with surgery alone and those receiving perioperative therapy plus surgery.

## Materials and methods

### Patients

From June 2005 to November 2022, 156 patients with LS-SCEC who underwent esophagectomy were included in this study. The study was registered with Chictr.org.cn (ChiCTR2400091485) and received approval from the Institutional Review Board (approval: SCCHEC-02-2023-130; approved November 15, 2023), which waived the requirement for individual written informed consent due to its retrospective design. The study adhered to the principles of the 1995 Declaration of Helsinki.

All patients met the following inclusion criteria: (1) preoperative or postoperative pathological confirmation of thoracic or abdominal SCEC; (2) preoperative evaluation indicating a limited stage without distant metastases (cTanyNanyM0) as defined by the 8th American Joint Committee on Cancer (AJCC) tumor, node and metastasis (TNM) classification^9^; (3) received curative esophagectomy with lymphadenectomy; and (4) availability of complete medical records. Exclusion criteria were: (1) cervical SCEC; (2) lack of medical or follow-up records; and (3) didn’t receive curative surgery.

Data collected included demographics, pathological findings, TNM staging, tumor location, neoadjuvant and adjuvant therapy details, surgical specifics, and survival times. All cases were restaged based on the 8th edition of the AJCC cancer staging manual.^9^ Pathological findings included SCEC type, lymph node involvement, vascular invasion, neural invasion, and immunohistochemical profiles.

### Treatment modalities

For patients who received neoadjuvant chemotherapy, etoposide combined with platinum was administered every 3 weeks for 2 to 3 cycles. For neoadjuvant radiotherapy, a concurrent radiation dose of 40.0 Gy was delivered in 20 fractions of 2.0 Gy over 5 days per week. Surgery was performed 3 to 4 weeks after neoadjuvant therapy completion. Surgical approaches included transthoracic esophagectomy or an abdominotranshiatal approach.

Postoperative treatments included adjuvant chemotherapy, radiotherapy, or chemoradiotherapy. Adjuvant chemotherapy consisted of 1 to 4 cycles of etoposide or irinotecan combined with platinum. Adjuvant radiotherapy involved a median dose of 60.0 Gy (range, 30-66 Gy) delivered in fractions of 1.8-2.0 Gy.

### Follow-up and outcome measurements

Patients were followed up every 3 months during the first 2 years, every 6 months for the subsequent 3 years, and annually thereafter. Follow-up was conducted via telephone or outpatient visits.

The primary endpoint, overall survival (OS), was defined as the time from initial treatment to death or last follow-up. ­Secondary endpoints included disease-free survival (DFS), measured from initial treatment to cancer recurrence or death, and postoperative complications. Complication severity was graded according to the Clavien–Dindo system,^10^ with grade IIIa or higher considered major complications.

Patients lost to follow-up were censored at the last contact date. The final general follow-up for survivors was conducted at the end of August 2023, with survivors censored on the last contact day.

### Statistical analysis

Continuous variables were presented as mean ± standard deviation, while categorical variables were expressed as number (%). Overall survival and DFS were calculated using the Kaplan-Meier method, and survival differences between groups were compared with the log-rank test. Multivariate analysis was performed using a stepwise Cox proportional hazards regression model. To balance baseline characteristics between patients with and without neoadjuvant chemotherapy, propensity score matching (PSM) was conducted in a 1:1 ratio using the nearest neighbor method without replacement. Variables included in the PSM analysis were surgical procedure (open vs minimally invasive), surgical approach (Sweet, Ivor-Lewis, McKeown, or transhiatal), preoperative pathological diagnosis, clinical N stage, and the number of dissected lymph nodes. All statistical tests were 2-sided, with *P* values <.05 considered statistically significant. Analyses were conducted using SPSS 24.0 (SPSS, Chicago, IL,USA) and R version 4.4.1 (2024-06-14).

## Results

### Patient characteristics

After screening 280 consecutive patients with SCEC, 156 were included in the study (Table 1, Figure S1). The median age was 62 years (interquartile range, 56-67), with a predominance of males (76.9% vs 23.1%). The mean number of lymph nodes removed was 19.0 ± 11.4. Of these, 33 (21.2%) patients received neoadjuvant chemotherapy, and 2 (1.3%) received neoadjuvant chemoradiotherapy. The proportion of patients receiving neoadjuvant therapy increased over time (Figure S2). Only 7 patients (4.5%) achieved pathological complete remission following neoadjuvant therapy.

**Table 1.** Patients’ characteristics and univariate analysis of OS and DFS.

Factors	Number of patients (%)	mOS (months)	5-year OS (%)	*P*	mDFS (months)	5-year DFS (%)	*P*
**Gender**				.074			.178
** Male**	120 (76.9)	22.0	29.5		13.0	24.7	
** Female**	36 (23.1)	17.0	14.6		10.0	12.6	
**Age (years)**				.694			.663
** ≤60**	66 (42.3)	21.0	24.2		11.0	22.1	
** >60**	90 (57.7)	21.0	26.9		12.0	21.9	
**Drinking**				.645			.773
** No**	75 (48.1)	21.0	26.1		12.0	22.2	
** Yes**	81 (51.9)	21.0	25.2		12.0	21.5	
**Smoking**				.506			.655
** No**	59 (37.8)	21.0	24.9		9.0	21.0	
** Yes**	97 (62.2)	22.0	26.0		13.0	21.7	
**Diet**				.072			.984
** Normal**	73 (46.8)	26.0	29.1		12.0	20.4	
** Abnormal**	83 (53.2)	17.0	22.6		12.0	23.1	
**Tumor location**				.172			.516
** Upper**	30 (19.2)	16.0	16.2		12.0	14.9	
** Middle**	66 (42.3)	20.0	25.8		12.0	20.9	
** Lower**	47 (30.1)	33.0	32.4		13.0	27.4	
** Esophagogastric junction**	13 (8.3)	16.0	23.1		8.0	23.1	
**Clinical T stage**				.011			<.001
** cT1**	26 (16.7)	44.0	39.2		23.0	39.3	
** cT2**	42 (26.9)	25.0	26.8		12.0	20.7	
** cT3**	78 (50.0)	17.0	23.5		12.0	19.7	
** cT4**	10 (6.4)	8.0	10.0		5.0	0.0	
**Clinical N stage**				.009			.004
** cN0**	86 (55.1)	24.0	32.2		13.0	30.0	
** cN+**	70 (44.9)	15.0	17.8		11.0	11.1	
**Surgical approach**				.925			.680
** Ivor-Lewis**	57 (36.5)	26.0	33.2		13.0	28.5	
** Mckewon**	86 (55.1)	19.0	23.1		12.0	19.2	
** Sweet**	8 (5.1)	24.0	14.6		8.0	12.5	
** Transhiatal**	5 (3.2)	15.0	20.0		5.0	20.0	
**Surgical procedure**				.313			.876
** Open**	95 (60.9)	19.0	25.4		12.0	23.3	
** MIE**	61 (39.1)	23.0	26.0		12.0	19.9	
**Neoadjuvant therapy**				.113^a^			.449^a^
** No**	121 (77.6)	19.0	23.0		12.0	20.5	
** Chemotherapy**	33 (21.2)	24.0	36.5		12.0	26.1	
** Chemoradiotherapy**	2 (1.3)	-	-		-	-	
**Adjuvant therapy**				<.001			<.001
** No**	65 (41.7)	12.0	15.6		9.0	10.4	
** Chemotherapy**	76 (48.7)	26.0	29.7		13.0	26.2	
** Radiotherapy**	2 (1.3)	-	-		-	-	
** Chemoradiotherapy**	13 (8.3)	65.0	56.4		NA	50.8	
**Pathologic T stage**				.027			.129
** pT0**	10 (6.4)	78.0	56.0		16.0	33.3	
** pT1-2**	75 (48.1)	24.0	30.4		12.0	26.2	
** pT3-4**	71 (45.5)	16.0	17.6		11.0	15.5	
**Pathologic N stage**				.001			.136
** pN0**	65 (41.7)	25.0	39.3		14.0	28.8	
** pN1**	45 (28.8)	21.0	18.5		9.0	19.3	
** pN2**	32 (20.5)	16.0	18.1		12.0	18.2	
** pN3**	14 (9.0)	12.0	7.1		12.0	7.8	
**Pathologic stage**				.002			.109
** I**	33 (21.2)	72.0	50.8		17.0	36.0	
** II**	40 (25.6)	22.0	23.7		12.0	18.0	
** III**	61 (39.1)	21.0	22.2		12.0	21.6	
** IVA**	22 (14.1)	12.0	9.6		12.0	10.2	
**Margin status**				.238			.572
** R0**	149 (95.5)	21.0	26.5		12.0	22.3	
** R1/2**	7 (4.5)	17.0	14.3		16.0	14.3	
**Vascular invasion**				.607			.853
** No**	114 (73.1)	21.0	23.9		12.0	19.9	
** Yes**	42 (26.9)	21.0	31.7		11.0	26.6	
**Neural invasion**				.001			.016
** No**	135 (86.5)	23.0	28.2		13.0	23.8	
** Yes**	21 (13.5)	12.0	11.5		8.0	10.0	
**Pathologic type**				.488			.888
** Pure small cell**	125 (80.1)	21.0	24.3		12.0	21.6	
** Mixed type**	31 (19.9)	19.0	32.7		12.0	22.6	
**Number of resected lymph node**				.457			.889
** <15**	61 (39.1)	24.0	26.3		12.0	22.0	
** ≥15**	95 (60.9)	19.0	26.2		12.0	22.0	

a Neoadjuvant chemotherapy vs direct surgery.

Abbreviations: mDFS, median disease-free survival; mOS, median overall survival; MIE, minimally invasive esophagectomy.

Adjuvant therapy included chemotherapy in 76 patients (48.7%), radiotherapy in 2 (1.3%), and chemoradiotherapy in 13 (8.3%). Of the 35 patients who received neoadjuvant chemotherapy, 11 did not receive any adjuvant therapy, 18 received adjuvant chemotherapy, 1 received adjuvant radiotherapy, and 5 received adjuvant chemoradiotherapy. Lymph node metastases were identified in 91 patients (58.3%) post-surgery. According to the 8th AJCC staging system for esophageal squamous cell carcinoma, 33 patients (21.2%) were classified as stage I post-surgery, 40 (25.6%) as stage II, 61 (39.1%) as stage III, and 22 (14.1%) as stage IVA.

### Postoperative complications

Postoperative complications occurred in 52 patients (33.3%), including 14 cases (9.0%) of major complications. Two patients (1.3%) died postoperatively, one from cerebral infarction and the other from pneumonia. Pneumonia was observed in 27 patients (17.3%) and anastomotic leakage in 16 (10.3%).

Following PSM analysis, 64 patients were matched—32 in the neoadjuvant chemotherapy group and 32 in the direct surgery group. No significant differences were observed between groups in overall postoperative complications (34.4% vs 37.5%, *P* = .794) or major complications (9.4% vs 12.5%, *P* = 1.000). Additionally, neoadjuvant chemotherapy did not increase the morbidity of pneumonia (25.0% vs 25.0%, *P* = 1.000) or anastomotic leakage (6.3% vs 12.5%, *P* = .672).

### Survival and recurrence analysis

By August 2023, with a median follow-up of 72 months (95% CI, 56.7-87.3 months), 109 patients (69.9%) had died. The median DFS and OS were 12.0 months (95% CI, 10.2-13.8 months) and 21.0 months (95% CI, 16.2-25.8 months), respectively. The 5-year OS and DFS rates were 25.9% and 21.9%, respectively (Figure S3).

A total of 67 patients (42.9%) had identifiable recurrence sites, including 42 with lymph node metastases, 25 with liver metastases, 13 with pulmonary metastases, 12 with bone metastases, 7 with brain metastases, 3 with pleural metastases, and 1 each with pancreatic, adrenal, and anastomotic site metastases. Recurrence patterns included locoregional in 8 patients (5.1%), distant recurrence in 30 (19.2%), and both locoregional and distant recurrence in 29 (18.6%).

### Univariate and multivariate analyses of prognostic factors

**Figure 1.**
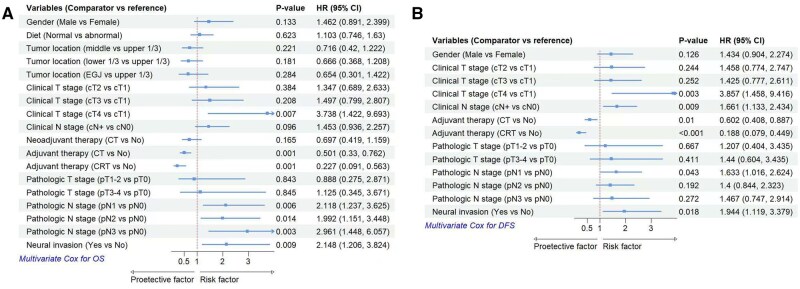
Multivariate Cox hazard ratios (HRs) for overall survival (OS) (A) and disease-free survival (DFS) (B) in limited-stage small cell esophageal carcinoma (LS-SCEC). CI, confidence intervals; CRT, chemoradiotherapy; CT, chemotherapy.

Univariate analysis identified clinical T stage (*P* = .011), clinical N stage (*P* = .009), pathological T stage (*P* = .027), pathological N stage (*P* = .001), pathological TNM stage (*P* = .002), adjuvant therapy (*P* < .001), and neural invasion (*P* = .001) as significant prognostic factors for OS (Table 1). Multivariate analysis further determined clinical T stage (*P* = .049), neural invasion (*P* = .002), N stage (*P* = .006), and adjuvant therapy (*P* = .001) as independent prognostic factors for OS (Figure 1).

For DFS, univariate analysis revealed that clinical T stage (*P* < .001), clinical N stage (*P* = .004), adjuvant therapy (*P* < .001), and neural invasion (*P* = .016) were significant prognostic factors (Table 1). Multivariate analysis confirmed clinical N stage (*P* = .009), adjuvant therapy (*P* < .001), and neural invasion (*P* = .018) as independent prognostic factors for DFS (Figure 1).

### The value of neoadjuvant chemotherapy after PSM

After PSM, the neoadjuvant chemotherapy and direct surgery groups each included 32 patients, with well-balanced baseline clinical characteristics (Table 2).

**Table 2.** Clinical and tumor characteristics of patients before and after propensity score matching.

Characteristic	No. (%) (Before matching)				No. (%) (After matching)			
	Overall (*n* = 154)	Neoadjuvant chemotherapy (*n* = 33)	Direct surgery (*n* = 121)	*P*	Overall (*n* = 64)	Neoadjuvant chemotherapy (*n* = 32)	Direct surgery (*n* = 32)	*P*
**Gastroscopic biopsy pathological type**				<.001				1.000^a^
** Small cell carcinoma**	85 (55.2)	29 (87.9)	56 (46.3)		56 (87.5)	28 (87.5)	28 (87.5)	
** Non-small cell carcinoma**	69 (44.8)	4 (12.1)	65 (53.7)		8 (12.5)	4 (12.5)	4 (12.5)	
**Clinical N stage**								.434
** cN0**	85 (55.2)	13 (39.4)	72 (59.5)	.039	23 (35.9)	13 (40.6)	10 (31.3)	
** cN+**	69 (44.8)	20 (60.6)	49 (40.5)		41 (64.1)	19 (59.4)	22 (68.7)	
**Surgical approach**				.041^a^				.492^a^
** Ivor-Lewis**	56 (36.4)	7 (21.2)	49 (40.5)		17 (26.6)	7 (21.9)	10 (31.3)	
** Mckewon**	85 (55.2)	25 (75.8)	60 (49.6)		45 (70.2)	24 (75.0)	21 (65.6)	
** Sweet**	8 (5.2)	0 (0)	8 (6.6)		1 (1.6)	0 (0.0)	1 (3.1)	
** Transhiatal**	5 (3.2)	1 (3.0)	4 (3.3)		1 (1.6)	1 (3.1)	0 (0.0)	
**Surgical procedure**				<.001				.045
** Open**	94 (61.0)	11 (33.3)	83 (68.6)		30 (46.9)	11 (34.4)	19 (59.4)	
** MIE**	60 (39.0)	22 (66.7)	38 (31.4)		34 (53.1)	21 (65.6)	13 (40.6)	
**Number of resected lymph node**				.018				.396
** <15**	60 (39.0)	7 (21.2)	53 (43.8)		17 (26.6)	7 (21.9)	10 (31.3)	
** ≥15**	94 (61.0)	26 (78.8)	68 (56.2)		47 (73.4)	25 (78.1)	22 (68.8)	
**Number of resected lymph node station**				.015				.396
** <6**	66 (42.9)	8 (24.2)	58 (47.9)		17 (26.6)	7 (21.9)	10 (31.3)	
** ≥6**	88 (57.1)	25 (75.8)	63 (52.1)		47 (73.4)	25 (78.1)	22 (68.8)	

a Fisher’s exact test.

Abbreviation: MIE, minimally invasive esophagectomy.

**Figure 2.**
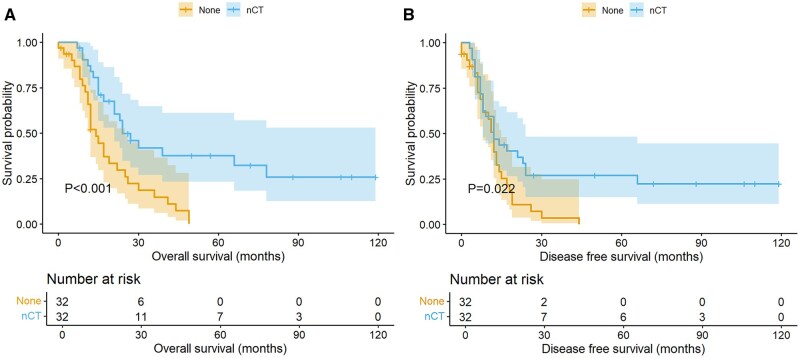
Kaplan-Meier survival curves of patients following propensity score-matched analysis, comparing OS (A) and DFS (B) for patients with and without neoadjuvant chemotherapy (nCT).

Both univariate and multivariate analyses demonstrated that neoadjuvant chemotherapy significantly improved OS in LS-SCEC patients, with a 5-year OS rate of 37.7% compared to 0.0% in the direct surgery group (*P* < .001, hazard ratio [HR] = 0.319, 95% CI, 0.168-0.604, Figure 2). The median OS was 24.0 months in the neoadjuvant chemotherapy group vs 14.0 months in the direct surgery group. Univariate analysis also found that neoadjuvant chemotherapy improved DFS in LS-SCEC patients (*P* = .022). However, multivariate analysis showed no significant improvement in DFS (*P* = .102, HR = 0.574, 95% CI, 0.295-1.117).

### Subgroup analysis for adjuvant therapy

**Figure 3.**
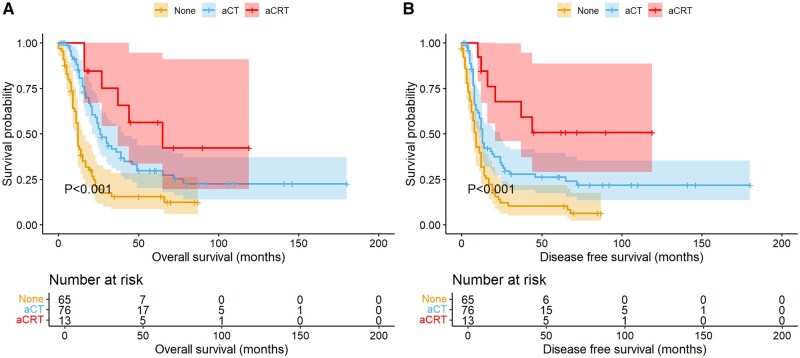
Kaplan-Meier survival curves for different adjuvant treatment modalities. The OS (A) and DFS (B) survival curves for the 3 groups. aCT, adjuvant chemotherapy; aCRT, adjuvant chemoradiotherapy.

In the overall cohort, adjuvant therapy improved both OS and DFS in LS-SCEC patients (Table 1, Figures 1 and 3). However, adjuvant chemoradiotherapy did not significantly improve OS compared to adjuvant chemotherapy alone (median OS: 65 vs 26 months; 5-year OS rate: 56.4% vs 29.7%; *P* = .101). It did, however, enhance DFS compared to chemotherapy alone (median DFS: not achieved vs 13 months; 5-year DFS rate: 50.8% vs 26.2%; *P* = 0.030). To further explore the value of adjuvant therapy, subgroup analyses were conducted based on postoperative pathological findings.

In the pT1-2 group, 30 patients didn’t receive adjuvant therapy, and 38 patients received adjuvant chemotherapy. Adjuvant chemotherapy improved OS (median OS: 30 vs 12 months; 5-year OS rate: 31.1% vs 22.4%; *P* = .042) but did not significantly impact DFS (median DFS: 13 vs 9 months; 5-year DFS rate: 29.7% vs 13.3%; *P* = .054). In the pT3-4 group, 33 patients didn’t receive adjuvant therapy, and 32 patients received adjuvant chemotherapy. Adjuvant chemotherapy improved both DFS (median DFS: 12 vs 7 months; 5-year DFS rate: 17.8% vs 6.5%; *P* = .012) and OS (median OS: 25 vs 12 months; 5-year OS rate: 21.6% vs 6.4%; *P* < .001).

In the pN0 group, 24 patients who didn’t receive adjuvant therapy had a median OS of 19.0 months and a 5-year OS rate of 33.1%. And 38 patients who received adjuvant chemotherapy had a median OS of 33.0 months, with a 5-year OS rate of 45.1% (*P *= .076). In the pN+ group, those without adjuvant therapy (*n* = 41) had a median OS of 12.0 months and a 5-year OS rate of 5.7%, whereas those receiving adjuvant chemotherapy (*n* = 38) had a median OS of 25.0 months and a 5-year OS rate of 13.8% (*P* = .001). Regardless of pathological N status, adjuvant chemotherapy consistently improved DFS (pN0 group: median DFS: 24.0 vs 9.0 months; 5-year DFS rate: 37.3% vs 16.7%; *P* = .029. pN+ group: median DFS: 12.0 vs 8.0 months; 5-year DFS rate: 14.7% vs 6.4%; *P* = .034).

For patients with pathological stage I-II, 26 didn’t receive adjuvant therapy, and 43 received adjuvant chemotherapy. Adjuvant chemotherapy improved both DFS (median DFS: 13 vs 9 months; 5-year DFS rate: 32.4% vs 11.5%; *P* = .013) and OS (median OS: 39 vs 17 months; 5-year OS rate: 38.6% vs 26.1%; *P* = .019). For patients with stage III-IV, 39 didn’t receive adjuvant therapy, and 33 received adjuvant chemotherapy. Adjuvant chemotherapy significantly improved OS (median OS: 21 vs 12 months; 5-year OS rate: 16.9% vs 9.1%; *P* = .010) and marginally improved DFS (median DFS: 12 vs 8 months; 5-year PFS rate: 17.4% vs 9.8%; *P* = .077). Furthermore, adding adjuvant radiotherapy (*n* = 9) to chemotherapy enhanced both OS (median OS: 65 vs 21 months; 5-year OS rate: 59.3% vs 16.9%; *P* = .030) and DFS (median DFS: Not available vs 12 months; 5-year DFS rate: 50.8% vs 17.4%; *P* = .017) (Figure S4).

## Discussion

Perioperative therapy plays a crucial role for patients with LS-SCEC. Previous studies with small sample sizes have not provided detailed long-term OS and DFS data following perioperative therapy. To the best of our knowledge, this study includes the largest cohort of LS-SCEC patients who underwent surgery at a single center, providing a comprehensive analysis of long-term outcomes after perioperative treatment. All the patients received surgery at one center reduced potential confounding factors. PSM analyses demonstrated that neoadjuvant chemotherapy significantly improved OS in LS-SCEC patients, while adjuvant therapy enhanced both OS and DFS.

There are notable differences in the choice of local therapy between China and the United States.^11^ In China, surgery is the predominant treatment, with 78.1% of patients undergoing surgical procedures, compared to only 8.1% in the United States, where radiotherapy is the primary local therapy. Xiao et al. suggested that surgery should be considered only for patients with localized disease (T1-4aN0M0). However, a recent large-scale, multicenter retrospective cohort study involving 458 LS-SCEC patients from 14 institutions in China indicated that surgery may be suitable for all LS-SCEC patients (TanyNanyM0).^12^ Both multivariate Cox regression and PSM analyses revealed no significant survival differences between the surgery and radiotherapy groups. Additionally, patients with tumors located in the lower thoracic esophagus or with tumor lengths exceeding 5 cm were more likely to benefit from esophagectomy as local treatment. Based on these findings, we advocate for surgery as the primary treatment for LS-SCEC patients, though further exploration of perioperative therapy within a multidisciplinary framework is warranted.

Cai et al. reported that neoadjuvant chemotherapy significantly reduced the risk of disease progression (HR 0.75; 95% CI, 0.57-0.99; *P* = .039) and mortality (HR 0.69; 95% CI, 0.51-0.92; *P* = .011), without increasing postoperative com­plication rates.^13^ Similarly, Xu et al. reviewed 75 stage III SCEC patients and found that neoadjuvant chemotherapy improved both OS and DFS compared to surgery alone.^14^ These findings align with our results. Based on the CROSS and NEOCRTEC5010 trials, neoadjuvant chemoradiotherapy has become the standard treatment for esophageal squamous cell carcinoma.^15^^,^^16^ In our study, only 2 patients received neoadjuvant chemoradiotherapy, one achieved pathological complete remission and remains alive at the last follow-up. Cai et al. noted that while neoadjuvant chemotherapy effectively reduced distant metastasis, it did not significantly lower the risk of locoregional metastasis.^13^ Other studies have suggested that chemoradiotherapy may provide a better prognosis than chemotherapy alone.^12^^,^^17^ These findings suggest the potential value of neoadjuvant chemoradiotherapy in LS-SCEC, warranting further investigation.

A recent small-scale retrospective study reported that adjuvant therapy did not significantly improve median OS in stage I-II patients but significantly benefited stage III patients.^18^ Conversely, Deng et al. found that adjuvant therapy significantly benefited stage I-II patients (*P* = .004) but not stage III patients (*P* = .136).^19^ These discrepancies may stem from small sample sizes and differences in TNM staging systems. Zou et al. conducted a retrospective, multicenter analysis of 150 LS-SCEC patients, categorizing them into limited stage I (LSI: T1-2N0M0) and LSII (T3–4N0M0 or T1–4N1–2M0) groups.^8^ They found that adjuvant therapy did not improve OS or DFS in LSI patients, but LSII patients receiving adjuvant chemotherapy experienced better OS and DFS. However, adding radiotherapy to adjuvant chemotherapy did not yield additional benefits. In this study, we found that adjuvant therapy generally improved outcomes for LS-SCEC patients. Those receiving adjuvant chemoradiotherapy had the longest OS and DFS among stage III-IV patients. Subgroup analyses revealed that adjuvant chemotherapy enhanced OS and DFS across most subgroups. However, the limited number of patients receiving adjuvant chemoradiotherapy restricted further subgroup analysis, highlighting the need for additional research.

This study has several limitations. First, as a retrospective analysis, it is subject to inherent biases and confounding factors. Multivariate and PSM analyses were employed to minimize these biases. Second, small sample sizes in certain subgroups, particularly those receiving neoadjuvant or adjuvant chemoradiotherapy, limited further analyses. These limitations should be considered when interpreting these results.

## Conclusion

In conclusion, perioperative therapy is crucial for LS-SCEC patients undergoing esophagectomy. Neoadjuvant chemotherapy improves OS in patients with LS-SCEC. Adjuvant chemotherapy is recommended almost for all patients with LS-SCEC. More clinical data are needed to conduct subgroup analysis and identify more effective systemic therapies for each patient with LS-SCEC.

## Supplementary Material

oyaf264_Supplementary_Data

## Data Availability

Further details and other data supporting the results of this study are available from the corresponding author upon request.
