# Supplementary material for: Perioperative therapy for limited-stage small cell esophageal carcinoma: a retrospective cohort study
Source: Oncologist. 2025 Aug 26;30(9):oyaf264. doi: 10.1093/oncolo/oyaf264 (PMC12448432; doi:10.1093/oncolo/oyaf264)
Supplement: oyaf264_Supplementary_Data [file oyaf264_supplementary_data.zip › Supplemental figure captions.docx]

**Supplemental figure captions**

Supplementary Figure 1：Diagram of patient selection.

Supplementary Figure 2: Treatment variations across the three different time periods. (A) Temporal trends in the use of neoadjuvant therapy. The proportion of patients receiving neoadjuvant therapy increased over time. DS: direct surgery, Nadj: neoadjuvant therapy. (B) Temporal trends in the use of adjuvant therapy. Adj: adjuvant therapy. (C) Temporal trends in the use of minimally invasive esophagectomy (MIE). The proportion of patients undergoing MIE increased over time. (D) Temporal trends in the use of different surgical approaches.

Supplementary Figure 3：Overall (A) and disease free (B) survival curves for the entire cohort.

Supplementary Figure 4：Kaplan-Meier survival curves of different adjuvant treatment modes in LS-SCEC patients with stage III-IV. aCT: adjuvant chemotherapy, aCRT: adjuvant chemoradiotherapy.
