# Supplementary figures and images for: Perioperative therapy for limited-stage small cell esophageal carcinoma: a retrospective cohort study
Source: Oncologist. 2025 Aug 26;30(9):oyaf264. doi: 10.1093/oncolo/oyaf264 (PMC12448432; doi:10.1093/oncolo/oyaf264)

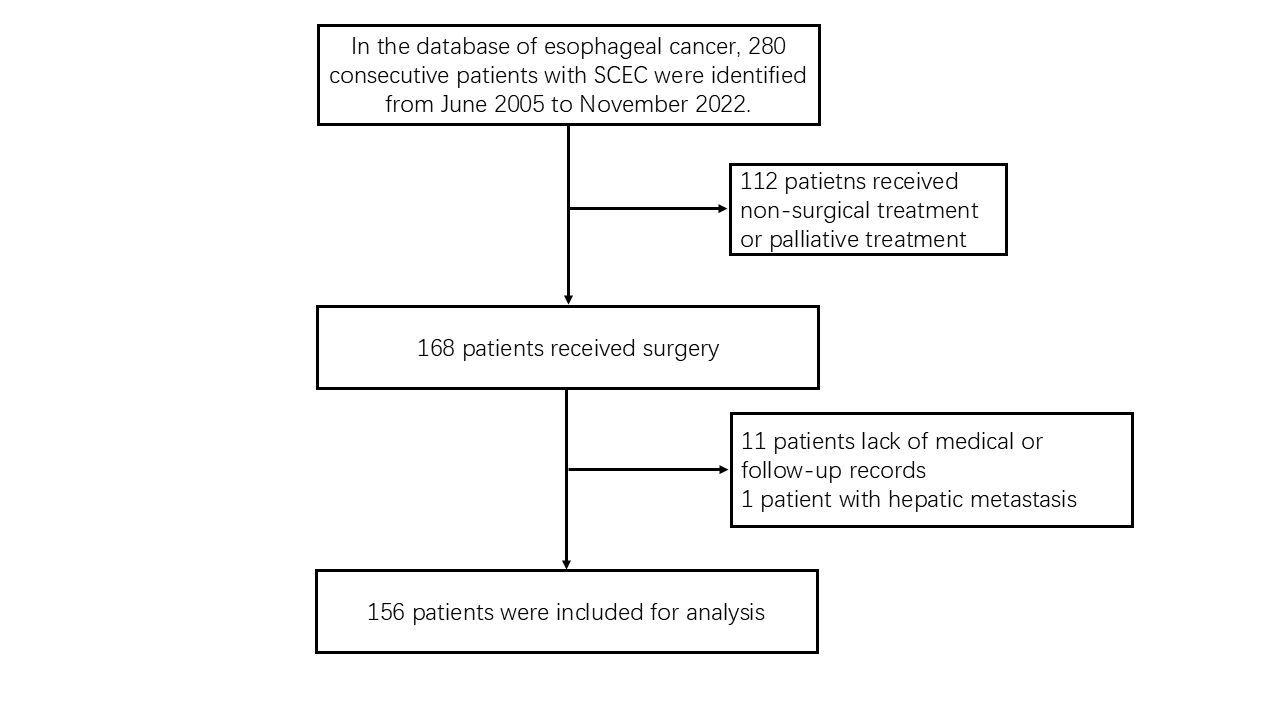

Supplement: oyaf264_Supplementary_Data [file oyaf264_supplementary_data.zip › Supplementary Figure 1.tif]

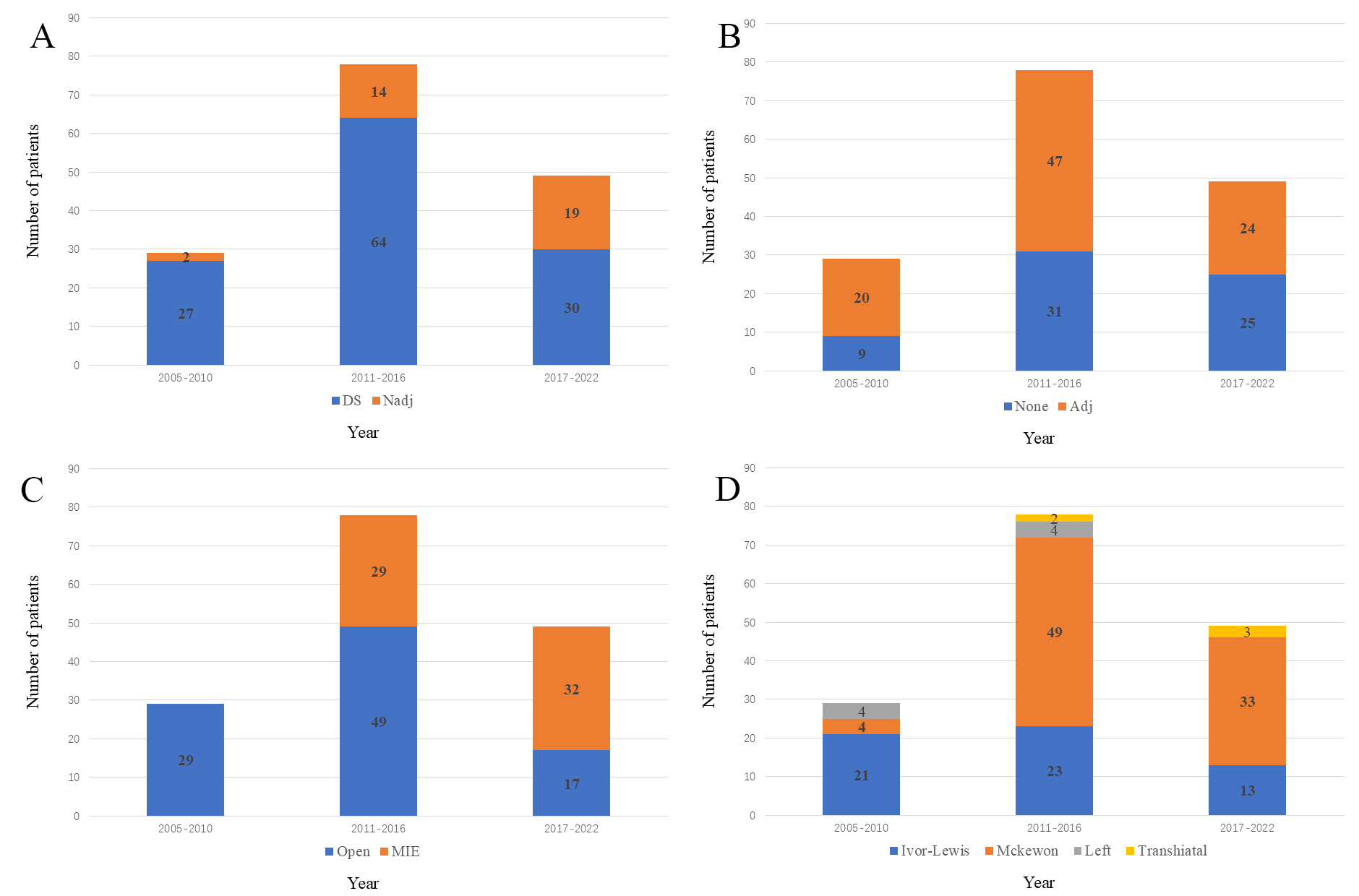

Supplement: oyaf264_Supplementary_Data [file oyaf264_supplementary_data.zip › Supplementary Figure 2.tif]

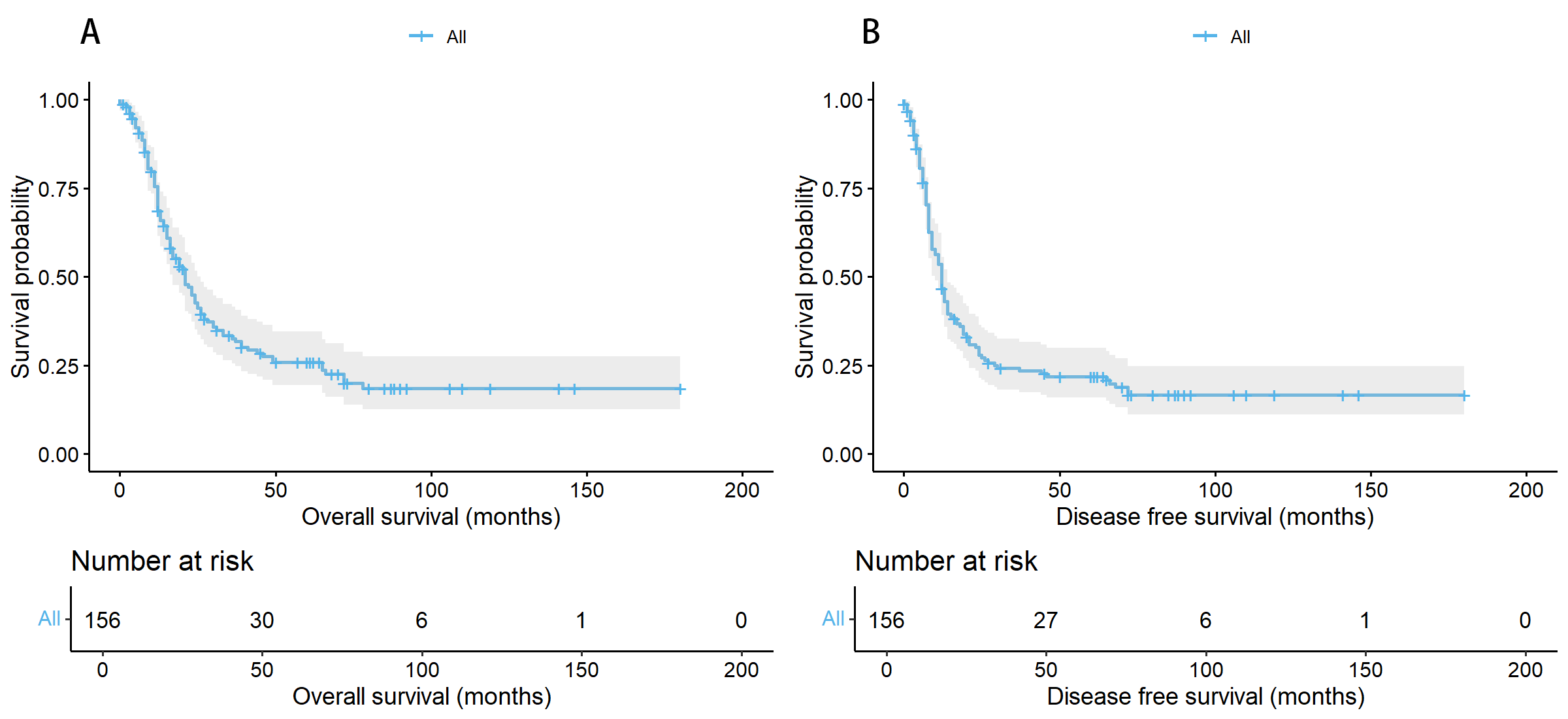

Supplement: oyaf264_Supplementary_Data [file oyaf264_supplementary_data.zip › Supplementary Figure 3.tif]

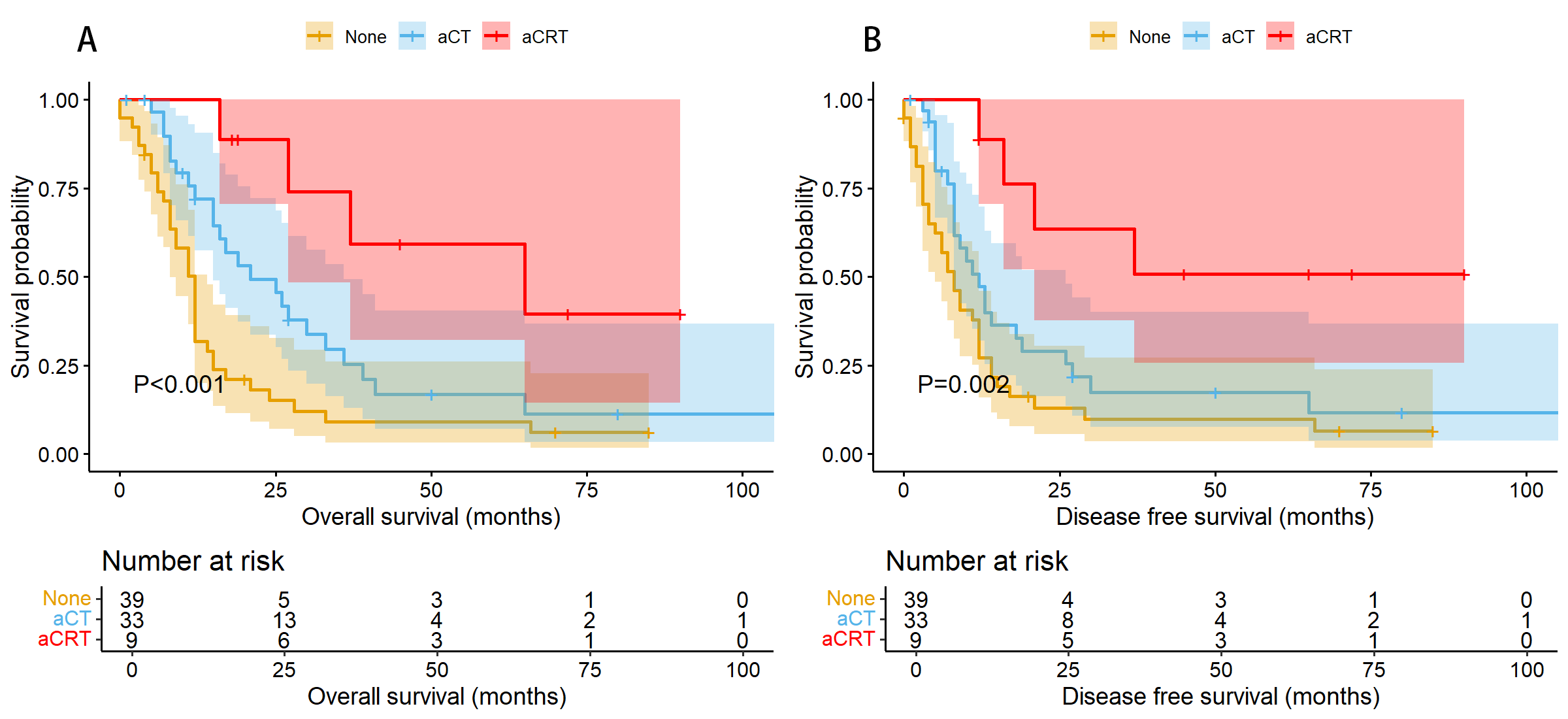

Supplement: oyaf264_Supplementary_Data [file oyaf264_supplementary_data.zip › Supplementary Figure 4.tif]
